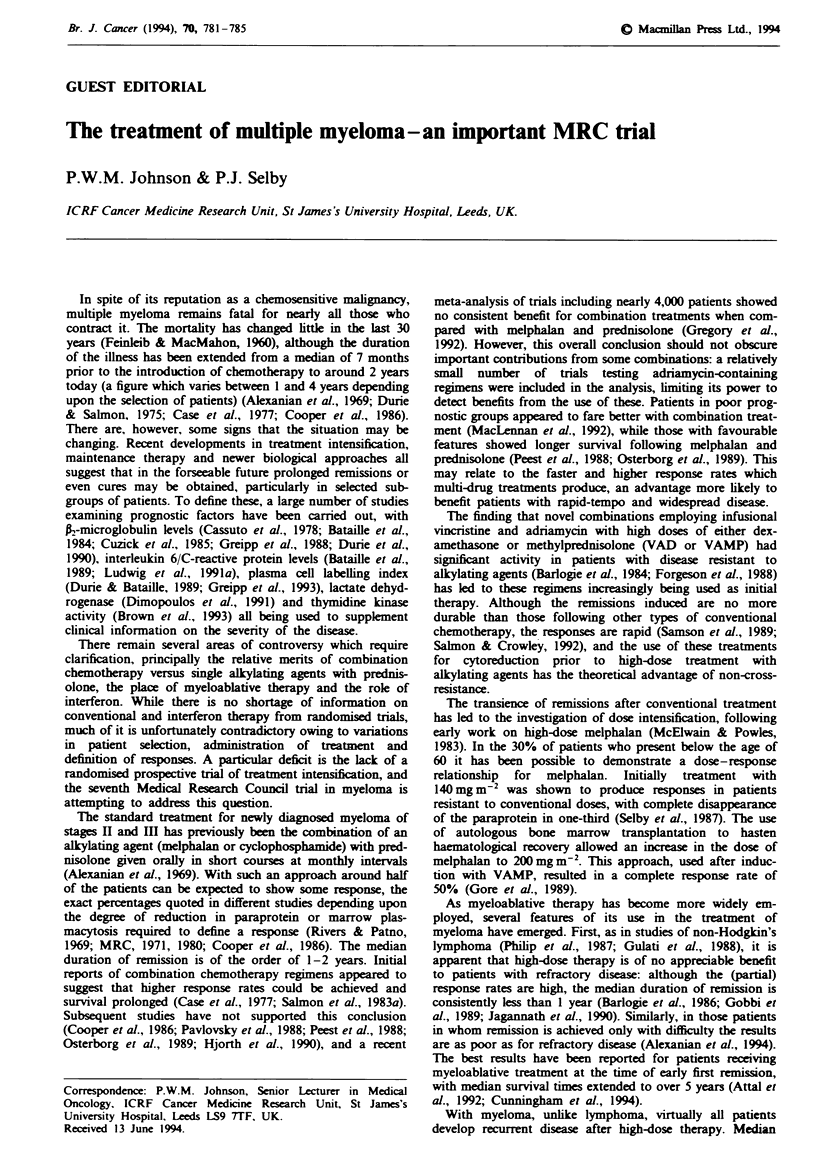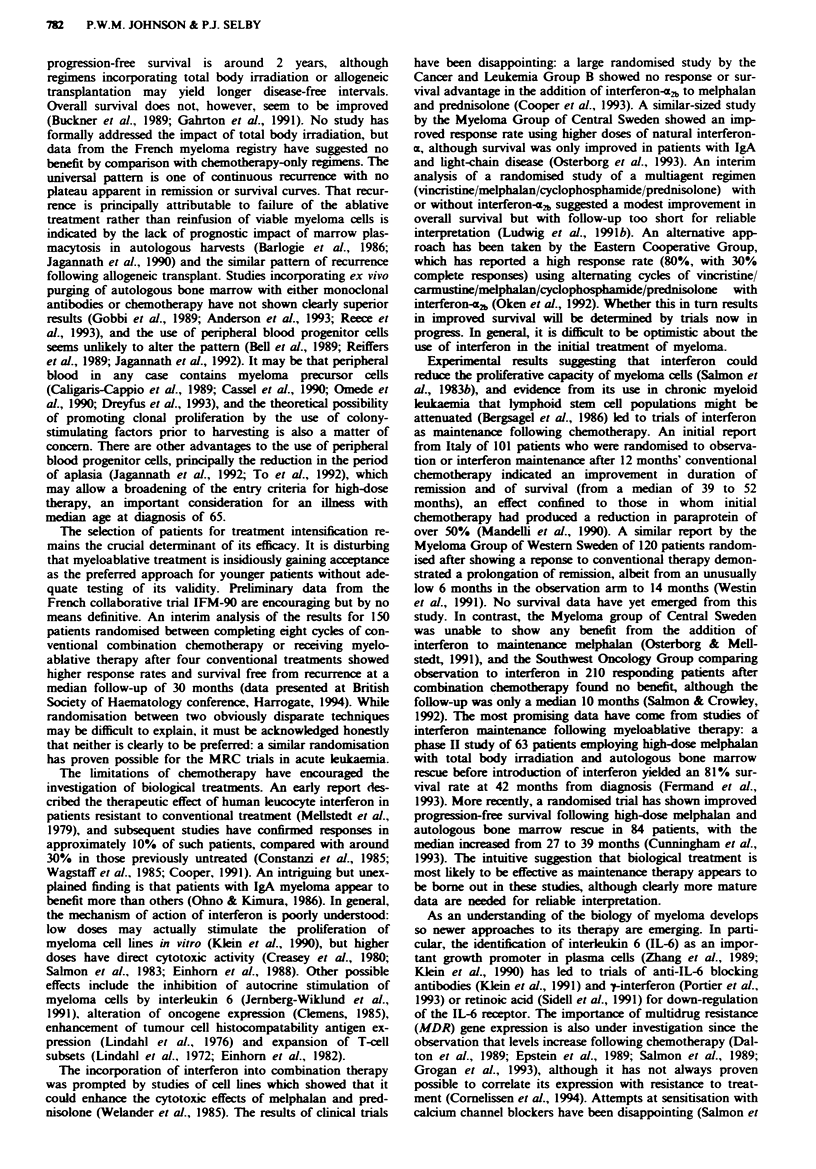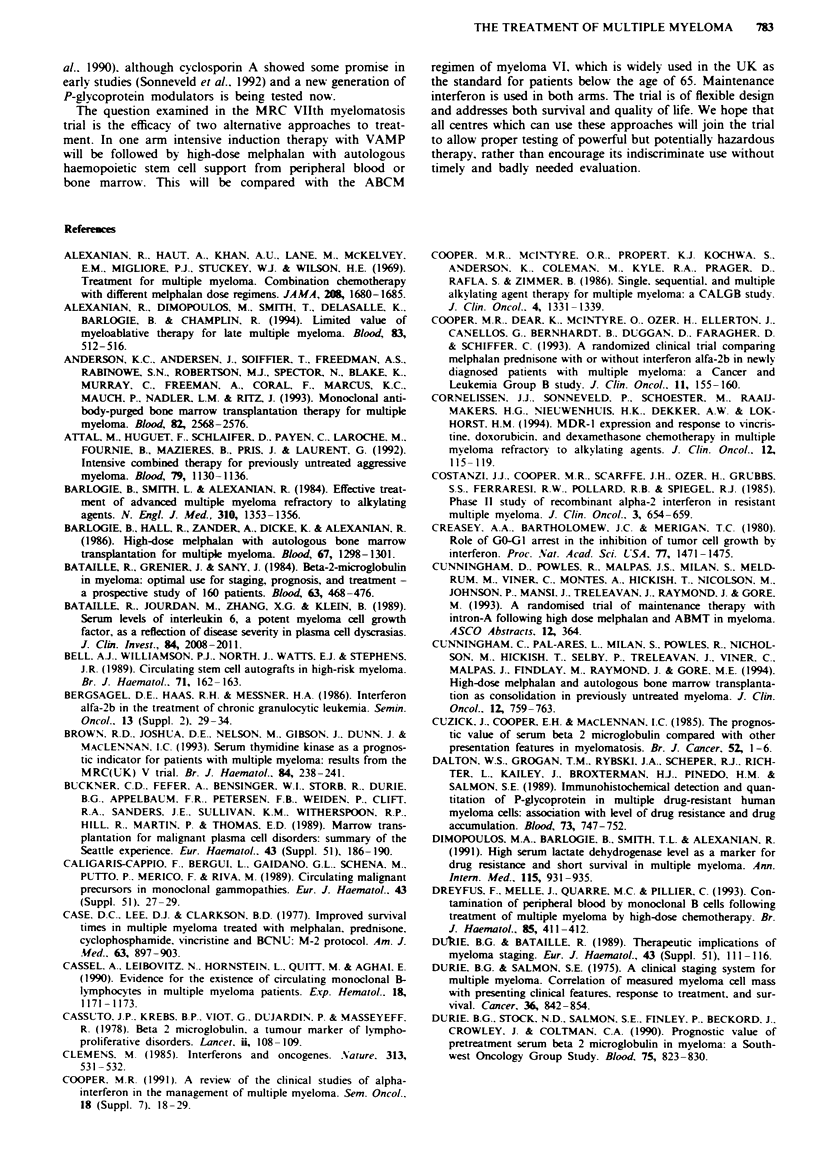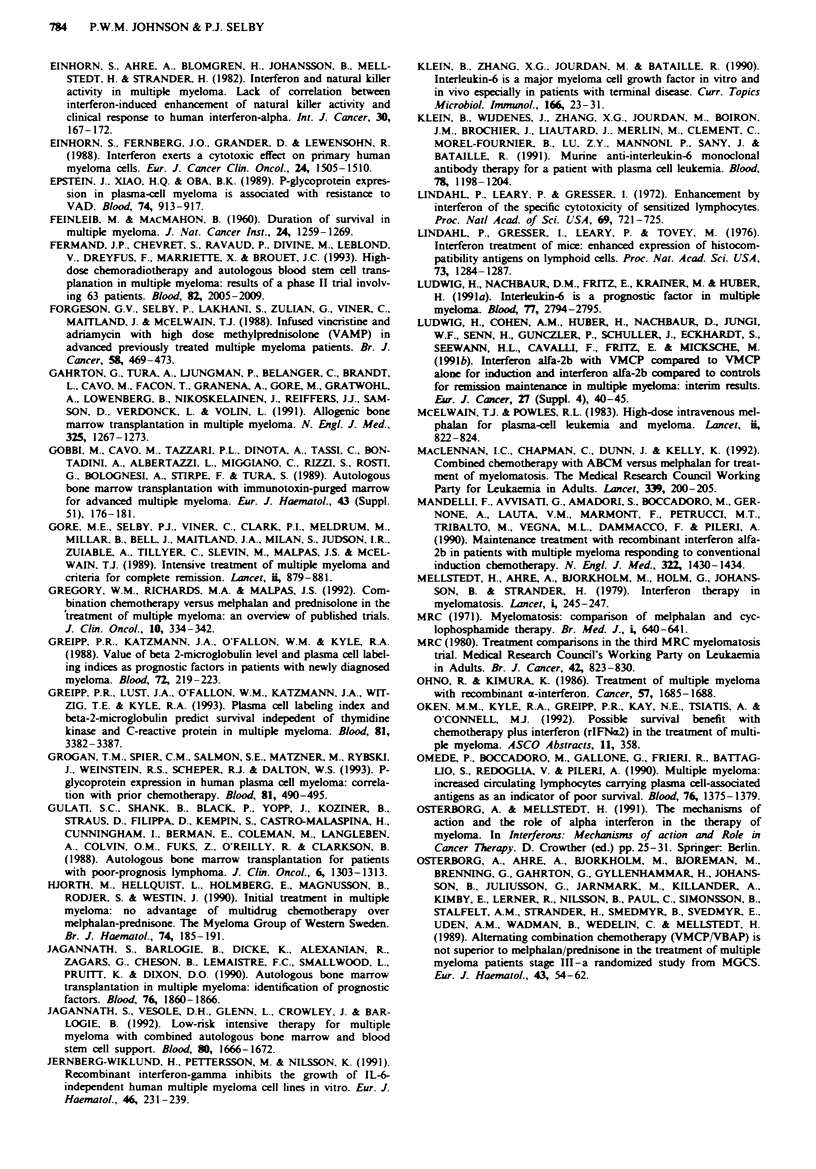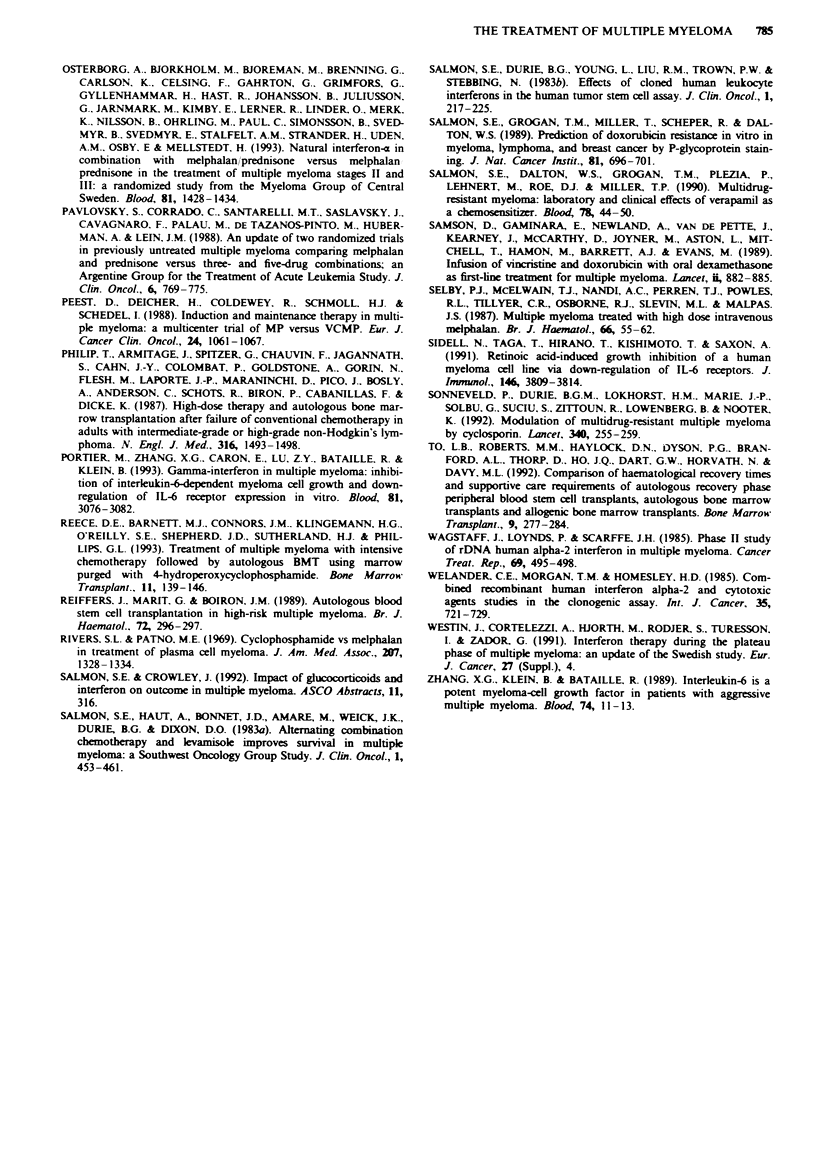# The treatment of multiple myeloma--an important MRC trial.

**DOI:** 10.1038/bjc.1994.399

**Published:** 1994-11

**Authors:** P. W. Johnson, P. J. Selby


GUEST EDITORIAL

### The treatment of multiple myeloma – an important MRC trial

P.W.M. Johnson & P.J. Selby

*ICRF Cancer Medicine Research Unit, St James's University Hospital, Leeds, UK.*

In spite of its reputation as a chemosensitive malignancy, multiple myeloma remains fatal for nearly all those who contract it. The mortality has changed little in the last 30 years (Feinleib & MacMahon, 1960), although the duration of the illness has been extended from a median of 7 months prior to the introduction of chemotherapy to around 2 years today (a figure which varies between 1 and 4 years depending upon the selection of patients) (Alexanian *et al*., 1969; Durie & Salmon, 1975; Case *et al*., 1977; Cooper *et al*., 1986). There are, however, some signs that the situation may be changing. Recent developments in treatment intensification, maintenance therapy and newer biological approaches all suggest that in the forseeable future prolonged remissions or even cures may be obtained, particularly in selected subgroups of patients. To define these, a large number of studies examining prognostic factors have been carried out, with $\beta_2$-microglobulin levels (Cassuto *et al*., 1978; Bataille *et al*., 1984; Cuzick *et al*., 1985; Greipp *et al*., 1988; Durie *et al*., 1990), interleukin 6/C-reactive protein levels (Bataille *et al*., 1989; Ludwig *et al*., 1991*a*), plasma cell labelling index (Durie & Bataille, 1989; Greipp *et al*., 1993), lactate dehydrogenase (Dimopoulos *et al*., 1991) and thymidine kinase activity (Brown *et al*., 1993) all being used to supplement clinical information on the severity of the disease.

There remain several areas of controversy which require clarification, principally the relative merits of combination chemotherapy versus single alkylating agents with prednisolone, the place of myeloablative therapy and the role of interferon. While there is no shortage of information on conventional and interferon therapy from randomised trials, much of it is unfortunately contradictory owing to variations in patient selection, administration of treatment and definition of responses. A particular deficit is the lack of a randomised prospective trial of treatment intensification, and the seventh Medical Research Council trial in myeloma is attempting to address this question.

The standard treatment for newly diagnosed myeloma of stages II and III has previously been the combination of an alkylating agent (melphalan or cyclophosphamide) with prednisolone given orally in short courses at monthly intervals (Alexanian *et al*., 1969). With such an approach around half of the patients can be expected to show some response, the exact percentages quoted in different studies depending upon the degree of reduction in paraprotein or marrow plasmacytosis required to define a response (Rivers & Patno, 1969; MRC, 1971, 1980; Cooper *et al*., 1986). The median duration of remission is of the order of 1-2 years. Initial reports of combination chemotherapy regimens appeared to suggest that higher response rates could be achieved and survival prolonged (Case *et al*., 1977; Salmon *et al*., 1983*a*). Subsequent studies have not supported this conclusion (Cooper *et al*., 1986; Pavlovsky *et al*., 1988; Peest *et al*., 1988; Osterborg *et al*., 1989; Hjorth *et al*., 1990), and a recent meta-analysis of trials including nearly 4,000 patients showed no consistent benefit for combination treatments when compared with melphalan and prednisolone (Gregory *et al*., 1992). However, this overall conclusion should not obscure important contributions from some combinations: a relatively small number of trials testing adriamycin-containing regimens were included in the analysis, limiting its power to detect benefits from the use of these. Patients in poor prognostic groups appeared to fare better with combination treatment (MacLennan *et al*., 1992), while those with favourable features showed longer survival following melphalan and prednisolone (Peest *et al*., 1988; Osterborg *et al*., 1989). This may relate to the faster and higher response rates which multi-drug treatments produce, an advantage more likely to benefit patients with rapid-tempo and widespread disease.

The finding that novel combinations employing infusional vincristine and adriamycin with high doses of either dexamethasone or methylprednisolone (VAD or VAMP) had significant activity in patients with disease resistant to alkylating agents (Barlogie *et al*., 1984; Forgeson *et al*., 1988) has led to these regimens increasingly being used as initial therapy. Although the remissions induced are no more durable than those following other types of conventional chemotherapy, the responses are rapid (Samson *et al*., 1989; Salmon & Crowley, 1992), and the use of these treatments for cytoreduction prior to high-dose treatment with alkylating agents has the theoretical advantage of non-cross-resistance.

The transience of remissions after conventional treatment has led to the investigation of dose intensification, following early work on high-dose melphalan (McElwain & Powles, 1983). In the 30% of patients who present below the age of 60 it has been possible to demonstrate a dose-response relationship for melphalan. Initially treatment with 140 mg $m^{-2}$ was shown to produce responses in patients resistant to conventional doses, with complete disappearance of the paraprotein in one-third (Selby *et al*., 1987). The use of autologous bone marrow transplantation to hasten haematological recovery allowed an increase in the dose of melphalan to 200 mg $m^{-2}$. This approach, used after induction with VAMP, resulted in a complete response rate of 50% (Gore *et al*., 1989).

As myeloablative therapy has become more widely employed, several features of its use in the treatment of myeloma have emerged. First, as in studies of non-Hodgkin's lymphoma (Philip *et al*., 1987; Gulati *et al*., 1988), it is apparent that high-dose therapy is of no appreciable benefit to patients with refractory disease: although the (partial) response rates are high, the median duration of remission is consistently less than 1 year (Barlogie *et al*., 1986; Gobbi *et al*., 1989; Jagannath *et al*., 1990). Similarly, in those patients in whom remission is achieved only with difficulty the results are as poor as for refractory disease (Alexanian *et al*., 1994). The best results have been reported for patients receiving myeloablative treatment at the time of early first remission, with median survival times extended to over 5 years (Attal *et al*., 1992; Cunningham *et al*., 1994).

With myeloma, unlike lymphoma, virtually all patients develop recurrent disease after high-dose therapy. Median

Correspondence: P.W.M. Johnson, Senior Lecturer in Medical Oncology, ICRF Cancer Medicine Research Unit, St James's University Hospital, Leeds LS9 7TF, UK.
Received 13 June 1994.

progression-free survival is around 2 years, although regimens incorporating total body irradiation or allogeneic transplantation may yield longer disease-free intervals. Overall survival does not, however, seem to be improved (Buckner *et al.*, 1989; Gahrton *et al.*, 1991). No study has formally addressed the impact of total body irradiation, but data from the French myeloma registry have suggested no benefit by comparison with chemotherapy-only regimens. The universal pattern is one of continuous recurrence with no plateau apparent in remission or survival curves. That recurrence is principally attributable to failure of the ablative treatment rather than reinfusion of viable myeloma cells is indicated by the lack of prognostic impact of marrow plasmacytosis in autologous harvests (Barlogie *et al.*, 1986; Jagannath *et al.*, 1990) and the similar pattern of recurrence following allogeneic transplant. Studies incorporating *ex vivo* purging of autologous bone marrow with either monoclonal antibodies or chemotherapy have not shown clearly superior results (Gobbi *et al.*, 1989; Anderson *et al.*, 1993; Reece *et al.*, 1993), and the use of peripheral blood progenitor cells seems unlikely to alter the pattern (Bell *et al.*, 1989; Reiffers *et al.*, 1989; Jagannath *et al.*, 1992). It may be that peripheral blood in any case contains myeloma precursor cells (Caligaris-Cappio *et al.*, 1989; Cassel *et al.*, 1990; Omede *et al.*, 1990; Dreyfus *et al.*, 1993), and the theoretical possibility of promoting clonal proliferation by the use of colony-stimulating factors prior to harvesting is also a matter of concern. There are other advantages to the use of peripheral blood progenitor cells, principally the reduction in the period of aplasia (Jagannath *et al.*, 1992; To *et al.*, 1992), which may allow a broadening of the entry criteria for high-dose therapy, an important consideration for an illness with median age at diagnosis of 65.

The selection of patients for treatment intensification remains the crucial determinant of its efficacy. It is disturbing that myeloablative treatment is insidiously gaining acceptance as the preferred approach for younger patients without adequate testing of its validity. Preliminary data from the French collaborative trial IFM-90 are encouraging but by no means definitive. An interim analysis of the results for 150 patients randomised between completing eight cycles of conventional combination chemotherapy or receiving myeloablative therapy after four conventional treatments showed higher response rates and survival free from recurrence at a median follow-up of 30 months (data presented at British Society of Haematology conference, Harrogate, 1994). While randomisation between two obviously disparate techniques may be difficult to explain, it must be acknowledged honestly that neither is clearly to be preferred: a similar randomisation has proven possible for the MRC trials in acute leukaemia.

The limitations of chemotherapy have encouraged the investigation of biological treatments. An early report described the therapeutic effect of human leucocyte interferon in patients resistant to conventional treatment (Mellstedt *et al.*, 1979), and subsequent studies have confirmed responses in approximately 10% of such patients, compared with around 30% in those previously untreated (Constanzi *et al.*, 1985; Wagstaff *et al.*, 1985; Cooper, 1991). An intriguing but unexplained finding is that patients with IgA myeloma appear to benefit more than others (Ohno & Kimura, 1986). In general, the mechanism of action of interferon is poorly understood: low doses may actually stimulate the proliferation of myeloma cell lines *in vitro* (Klein *et al.*, 1990), but higher doses have direct cytotoxic activity (Creasey *et al.*, 1980; Salmon *et al.*, 1983; Einhorn *et al.*, 1988). Other possible effects include the inhibition of autocrine stimulation of myeloma cells by interleukin 6 (Jernberg-Wiklund *et al.*, 1991), alteration of oncogene expression (Clemens, 1985), enhancement of tumour cell histocompatability antigen expression (Lindahl *et al.*, 1976) and expansion of T-cell subsets (Lindahl *et al.*, 1972; Einhorn *et al.*, 1982).

The incorporation of interferon into combination therapy was prompted by studies of cell lines which showed that it could enhance the cytotoxic effects of melphalan and prednisolone (Welander *et al.*, 1985). The results of clinical trials have been disappointing: a large randomised study by the Cancer and Leukemia Group B showed no response or survival advantage in the addition of interferon-$\alpha_{2b}$ to melphalan and prednisolone (Cooper *et al.*, 1993). A similar-sized study by the Myeloma Group of Central Sweden showed an improved response rate using higher doses of natural interferon-$\alpha$, although survival was only improved in patients with IgA and light-chain disease (Osterborg *et al.*, 1993). An interim analysis of a randomised study of a multiagent regimen (vincristine/melphalan/cyclophosphamide/prednisolone) with or without interferon-$\alpha_{2b}$ suggested a modest improvement in overall survival but with follow-up too short for reliable interpretation (Ludwig *et al.*, 1991*b*). An alternative approach has been taken by the Eastern Cooperative Group, which has reported a high response rate (80%, with 30% complete responses) using alternating cycles of vincristine/carmustine/melphalan/cyclophosphamide/prednisolone with interferon-$\alpha_{2b}$ (Oken *et al.*, 1992). Whether this in turn results in improved survival will be determined by trials now in progress. In general, it is difficult to be optimistic about the use of interferon in the initial treatment of myeloma.

Experimental results suggesting that interferon could reduce the proliferative capacity of myeloma cells (Salmon *et al.*, 1983*b*), and evidence from its use in chronic myeloid leukaemia that lymphoid stem cell populations might be attenuated (Bergsagel *et al.*, 1986) led to trials of interferon as maintenance following chemotherapy. An initial report from Italy of 101 patients who were randomised to observation or interferon maintenance after 12 months' conventional chemotherapy indicated an improvement in duration of remission and of survival (from a median of 39 to 52 months), an effect confined to those in whom initial chemotherapy had produced a reduction in paraprotein of over 50% (Mandelli *et al.*, 1990). A similar report by the Myeloma Group of Western Sweden of 120 patients randomised after showing a reponse to conventional therapy demonstrated a prolongation of remission, albeit from an unusually low 6 months in the observation arm to 14 months (Westin *et al.*, 1991). No survival data have yet emerged from this study. In contrast, the Myeloma group of Central Sweden was unable to show any benefit from the addition of interferon to maintenance melphalan (Osterborg & Mellstedt, 1991), and the Southwest Oncology Group comparing observation to interferon in 210 responding patients after combination chemotherapy found no benefit, although the follow-up was only a median 10 months (Salmon & Crowley, 1992). The most promising data have come from studies of interferon maintenance following myeloablative therapy: a phase II study of 63 patients employing high-dose melphalan with total body irradiation and autologous bone marrow rescue before introduction of interferon yielded an 81% survival rate at 42 months from diagnosis (Fermand *et al.*, 1993). More recently, a randomised trial has shown improved progression-free survival following high-dose melphalan and autologous bone marrow rescue in 84 patients, with the median increased from 27 to 39 months (Cunningham *et al.*, 1993). The intuitive suggestion that biological treatment is most likely to be effective as maintenance therapy appears to be borne out in these studies, although clearly more mature data are needed for reliable interpretation.

As an understanding of the biology of myeloma develops so newer approaches to its therapy are emerging. In particular, the identification of interleukin 6 (IL-6) as an important growth promoter in plasma cells (Zhang *et al.*, 1989; Klein *et al.*, 1990) has led to trials of anti-IL-6 blocking antibodies (Klein *et al.*, 1991) and $\gamma$-interferon (Portier *et al.*, 1993) or retinoic acid (Sidell *et al.*, 1991) for down-regulation of the IL-6 receptor. The importance of multidrug resistance (*MDR*) gene expression is also under investigation since the observation that levels increase following chemotherapy (Dalton *et al.*, 1989; Epstein *et al.*, 1989; Salmon *et al.*, 1989; Grogan *et al.*, 1993), although it has not always proven possible to correlate its expression with resistance to treatment (Cornelissen *et al.*, 1994). Attempts at sensitisation with calcium channel blockers have been disappointing (Salmon *et*

*al.*, 1990), although cyclosporin A showed some promise in early studies (Sonneveld *et al.*, 1992) and a new generation of *P*-glycoprotein modulators is being tested now.

The question examined in the MRC VIIth myelomatosis trial is the efficacy of two alternative approaches to treatment. In one arm intensive induction therapy with VAMP will be followed by high-dose melphalan with autologous haemopoietic stem cell support from peripheral blood or bone marrow. This will be compared with the ABCM regimen of myeloma VI, which is widely used in the UK as the standard for patients below the age of 65. Maintenance interferon is used in both arms. The trial is of flexible design and addresses both survival and quality of life. We hope that all centres which can use these approaches will join the trial to allow proper testing of powerful but potentially hazardous therapy, rather than encourage its indiscriminate use without timely and badly needed evaluation.

### References

ALEXANIAN, R., HAUT, A., KHAN, A.U., LANE, M., MCKELVEY, E.M., MIGLIORE, P.J., STUCKEY, W.J. & WILSON, H.E. (1969). Treatment for multiple myeloma. Combination chemotherapy with different melphalan dose regimens. *JAMA*, **208**, 1680-1685.

ALEXANIAN, R., DIMOPOULOS, M., SMITH, T., DELASALLE, K., BARLOGIE, B. & CHAMPLIN, R. (1994). Limited value of myeloablative therapy for late multiple myeloma. *Blood*, **83**, 512-516.

ANDERSON, K.C., ANDERSEN, J., SOIFFIER, T., FREEDMAN, A.S., RABINOWE, S.N., ROBERTSON, M.J., SPECTOR, N., BLAKE, K., MURRAY, C., FREEMAN, A., CORAL, F., MARCUS, K.C., MAUCH, P., NADLER, L.M. & RITZ, J. (1993). Monoclonal antibody-purged bone marrow transplantation therapy for multiple myeloma. *Blood*, **82**, 2568-2576.

ATTAL, M., HUGUET, F., SCHLAIFER, D., PAYEN, C., LAROCHE, M., FOURNIE, B., MAZIERES, B., PRIS, J. & LAURENT, G. (1992). Intensive combined therapy for previously untreated aggressive myeloma. *Blood*, **79**, 1130-1136.

BARLOGIE, B., SMITH, L. & ALEXANIAN, R. (1984). Effective treatment of advanced multiple myeloma refractory to alkylating agents. *N. Engl. J. Med.*, **310**, 1353-1356.

BARLOGIE, B., HALL, R., ZANDER, A., DICKE, K. & ALEXANIAN, R. (1986). High-dose melphalan with autologous bone marrow transplantation for multiple myeloma. *Blood*, **67**, 1298-1301.

BATAILLE, R., GRENIER, J. & SANY, J. (1984). Beta-2-microglobulin in myeloma: optimal use for staging, prognosis, and treatment – a prospective study of 160 patients. *Blood*, **63**, 468-476.

BATAILLE, R., JOURDAN, M., ZHANG, X.G. & KLEIN, B. (1989). Serum levels of interleukin 6, a potent myeloma cell growth factor, as a reflection of disease severity in plasma cell dyscrasias. *J. Clin. Invest.*, **84**, 2008-2011.

BELL, A.J., WILLIAMSON, P.J., NORTH, J., WATTS, E.J. & STEPHENS, J.R. (1989). Circulating stem cell autografts in high-risk myeloma. *Br. J. Haematol.*, **71**, 162-163.

BERGSAGEL, D.E., HAAS, R.H. & MESSNER, H.A. (1986). Interferon alfa-2b in the treatment of chronic granulocytic leukemia. *Semin. Oncol.*, **13** (Suppl. 2), 29-34.

BROWN, R.D., JOSHUA, D.E., NELSON, M., GIBSON, J., DUNN, J. & MACLENNAN, I.C. (1993). Serum thymidine kinase as a prognostic indicator for patients with multiple myeloma: results from the MRC(UK) V trial. *Br. J. Haematol.*, **84**, 238-241.

BUCKNER, C.D., FEFER, A., BENSINGER, W.I., STORB, R., DURIE, B.G., APPELBAUM, F.R., PETERSEN, F.B., WEIDEN, P., CLIFT, R.A., SANDERS, J.E., SULLIVAN, K.M., WITHERSPOON, R.P., HILL, R., MARTIN, P. & THOMAS, E.D. (1989). Marrow transplantation for malignant plasma cell disorders: summary of the Seattle experience. *Eur. Haematol.*, **43** (Suppl. 51), 186-190.

CALIGARIS-CAPPIO, F., BERGUI, L., GAIDANO, G.L., SCHENA, M., PUTTO, P., MERICO, F. & RIVA, M. (1989). Circulating malignant precursors in monoclonal gammopathies. *Eur. J. Haematol.*, **43** (Suppl. 51), 27-29.

CASE, D.C., LEE, D.J. & CLARKSON, B.D. (1977). Improved survival times in multiple myeloma treated with melphalan, prednisone, cyclophosphamide, vincristine and BCNU: M-2 protocol. *Am. J. Med.*, **63**, 897-903.

CASSEL, A., LEIBOVITZ, N., HORNSTEIN, L., QUITT, M. & AGHAI, E. (1990). Evidence for the existence of circulating monoclonal B-lymphocytes in multiple myeloma patients. *Exp. Hematol.*, **18**, 1171-1173.

CASSUTO, J.P., KREBS, B.P., VIOT, G., DUJARDIN, P. & MASSEYEFF, R. (1978). Beta 2 microglobulin, a tumour marker of lymphoproliferative disorders. *Lancet*, **ii**, 108-109.

CLEMENS, M. (1985). Interferons and oncogenes. *Nature*, **313**, 531-532.

COOPER, M.R. (1991). A review of the clinical studies of alpha-interferon in the management of multiple myeloma. *Sem. Oncol.*, **18** (Suppl. 7), 18-29.

COOPER, M.R., MCINTYRE, O.R., PROPERT, K.J. KOCHWA, S., ANDERSON, K., COLEMAN, M., KYLE, R.A., PRAGER, D., RAFLA, S. & ZIMMER, B. (1986). Single, sequential, and multiple alkylating agent therapy for multiple myeloma: a CALGB study. *J. Clin. Oncol.*, **4**, 1331-1339.

COOPER, M.R., DEAR, K., MCINTYRE, O., OZER, H., ELLERTON, J., CANELLOS, G., BERNHARDT, B., DUGGAN, D., FARAGHER, D. & SCHIFFER, C. (1993). A randomized clinical trial comparing melphalan prednisone with or without interferon alfa-2b in newly diagnosed patients with multiple myeloma: a Cancer and Leukemia Group B study. *J. Clin. Oncol.*, **11**, 155-160.

CORNELISSEN, J.J., SONNEVELD, P., SCHOESTER, M., RAAIJMAKERS, H.G., NIEUWENHUIS, H.K., DEKKER, A.W. & LOKHORST, H.M. (1994). MDR-1 expression and response to vincristine, doxorubicin, and dexamethasone chemotherapy in multiple myeloma refractory to alkylating agents. *J. Clin. Oncol.*, **12**, 115-119.

COSTANZI, J.J., COOPER, M.R., SCARFFE, J.H., OZER, H., GRUBBS, S.S., FERRARESI, R.W., POLLARD, R.B. & SPIEGEL, R.J. (1985). Phase II study of recombinant alpha-2 interferon in resistant multiple myeloma. *J. Clin. Oncol.*, **3**, 654-659.

CREASEY, A.A., BARTHOLOMEW, J.C. & MERIGAN, T.C. (1980). Role of G0-G1 arrest in the inhibition of tumor cell growth by interferon. *Proc. Nat. Acad. Sci. USA*, **77**, 1471-1475.

CUNNINGHAM, D., POWLES, R., MALPAS, J.S., MILAN, S., MELDRUM, M., VINER, C., MONTES, A., HICKISH, T., NICOLSON, M., JOHNSON, P., MANSI, J., TRELEAVAN, J., RAYMOND, J. & GORE, M. (1993). A randomised trial of maintenance therapy with intron-A following high dose melphalan and ABMT in myeloma. *ASCO Abstracts*, **12**, 364.

CUNNINGHAM, C., PAL-ARES, L., MILAN, S., POWLES, R., NICHOLSON, M., HICKISH, T., SELBY, P., TRELEAVAN, J., VINER, C., MALPAS, J., FINDLAY, M., RAYMOND, J. & GORE, M.E. (1994). High-dose melphalan and autologous bone marrow transplantation as consolidation in previously untreated myeloma. *J. Clin. Oncol.*, **12**, 759-763.

CUZICK, J., COOPER, E.H. & MACLENNAN, I.C. (1985). The prognostic value of serum beta 2 microglobulin compared with other presentation features in myelomatosis. *Br. J. Cancer*, **52**, 1-6.

DALTON, W.S., GROGAN, T.M., RYBSKI, J.A., SCHEPER, R.J., RICHTER, L., KAILEY, J., BROXTERMAN, H.J., PINEDO, H.M. & SALMON, S.E. (1989). Immunohistochemical detection and quantitation of P-glycoprotein in multiple drug-resistant human myeloma cells: association with level of drug resistance and drug accumulation. *Blood*, **73**, 747-752.

DIMOPOULOS, M.A., BARLOGIE, B., SMITH, T.L. & ALEXANIAN, R. (1991). High serum lactate dehydrogenase level as a marker for drug resistance and short survival in multiple myeloma. *Ann. Intern. Med.*, **115**, 931-935.

DREYFUS, F., MELLE, J., QUARRE, M.C. & PILLIER, C. (1993). Contamination of peripheral blood by monoclonal B cells following treatment of multiple myeloma by high-dose chemotherapy. *Br. J. Haematol.*, **85**, 411-412.

DURIE, B.G. & BATAILLE, R. (1989). Therapeutic implications of myeloma staging. *Eur. J. Haematol.*, **43** (Suppl. 51), 111-116.

DURIE, B.G. & SALMON, S.E. (1975). A clinical staging system for multiple myeloma. Correlation of measured myeloma cell mass with presenting clinical features, response to treatment, and survival. *Cancer*, **36**, 842-854.

DURIE, B.G., STOCK, N.D., SALMON, S.E., FINLEY, P., BECKORD, J., CROWLEY, J. & COLTMAN, C.A. (1990). Prognostic value of pretreatment serum beta 2 microglobulin in myeloma: a Southwest Oncology Group Study. *Blood*, **75**, 823-830.

EINHORN, S., AHRE, A., BLOMGREN, H., JOHANSSON, B., MELLSTEDT, H. & STRANDER, H. (1982). Interferon and natural killer activity in multiple myeloma. Lack of correlation between interferon-induced enhancement of natural killer activity and clinical response to human interferon-alpha. *Int. J. Cancer*, **30**, 167-172.

EINHORN, S., FERNBERG, J.O., GRANDER, D. & LEWENSOHN, R. (1988). Interferon exerts a cytotoxic effect on primary human myeloma cells. *Eur. J. Cancer Clin. Oncol.*, **24**, 1505-1510.

EPSTEIN, J., XIAO, H.Q. & OBA, B.K. (1989). P-glycoprotein expression in plasma-cell myeloma is associated with resistance to VAD. *Blood*, **74**, 913-917.

FEINLEIB, M. & MACMAHON, B. (1960). Duration of survival in multiple myeloma. *J. Nat. Cancer Inst.*, **24**, 1259-1269.

FERMAND, J.P., CHEVRET, S., RAVAUD, P., DIVINE, M., LEBLOND, V., DREYFUS, F., MARRIETTE, X. & BROUET, J.C. (1993). High-dose chemoradiotherapy and autologous blood stem cell transplanation in multiple myeloma: results of a phase II trial involving 63 patients. *Blood*, **82**, 2005-2009.

FORGESON, G.V., SELBY, P., LAKHANI, S., ZULIAN, G., VINER, C., MAITLAND, J. & MCELWAIN, T.J. (1988). Infused vincristine and adriamycin with high dose methylprednisolone (VAMP) in advanced previously treated multiple myeloma patients. *Br. J. Cancer*, **58**, 469-473.

GAHRTON, G., TURA, A., LJUNGMAN, P., BELANGER, C., BRANDT, L., CAVO, M., FACON, T., GRANENA, A., GORE, M., GRATWOHL, A., LOWENBERG, B., NIKOSKELAINEN, J., REIFFERS, J.J., SAMSON, D., VERDONCK, L. & VOLIN, L. (1991). Allogenic bone marrow transplantation in multiple myeloma. *N. Engl. J. Med.*, **325**, 1267-1273.

GOBBI, M., CAVO, M., TAZZARI, P.L., DINOTA, A., TASSI, C., BONTADINI, A., ALBERTAZZI, L., MIGGIANO, C., RIZZI, S., ROSTI, G., BOLOGNESI, A., STIRPE, F. & TURA, S. (1989). Autologous bone marrow transplantation with immunotoxin-purged marrow for advanced multiple myeloma. *Eur. J. Haematol.*, **43** (Suppl. 51), 176-181.

GORE, M.E., SELBY, P.J., VINER, C., CLARK, P.I., MELDRUM, M., MILLAR, B., BELL, J., MAITLAND, J.A., MILAN, S., JUDSON, I.R., ZUIABLE, A., TILLYER, C., SLEVIN, M., MALPAS, J.S. & MCELWAIN, T.J. (1989). Intensive treatment of multiple myeloma and criteria for complete remission. *Lancet*, **ii**, 879-881.

GREGORY, W.M., RICHARDS, M.A. & MALPAS, J.S. (1992). Combination chemotherapy versus melphalan and prednisolone in the treatment of multiple myeloma: an overview of published trials. *J. Clin. Oncol.*, **10**, 334-342.

GREIPP, P.R., KATZMANN, J.A., O'FALLON, W.M. & KYLE, R.A. (1988). Value of beta 2-microglobulin level and plasma cell labeling indices as prognostic factors in patients with newly diagnosed myeloma. *Blood*, **72**, 219-223.

GREIPP, P.R., LUST, J.A., O'FALLON, W.M., KATZMANN, J.A., WITZIG, T.E. & KYLE, R.A. (1993). Plasma cell labeling index and beta-2-microglobulin predict survival indepedent of thymidine kinase and C-reactive protein in multiple myeloma. *Blood*, **81**, 3382-3387.

GROGAN, T.M., SPIER, C.M., SALMON, S.E., MATZNER, M., RYBSKI, J., WEINSTEIN, R.S., SCHEPER, R.J. & DALTON, W.S. (1993). P-glycoprotein expression in human plasma cell myeloma: correlation with prior chemotherapy. *Blood*, **81**, 490-495.

GULATI, S.C., SHANK, B., BLACK, P., YOPP, J., KOZINER, B., STRAUS, D., FILIPPA, D., KEMPIN, S., CASTRO-MALASPINA, H., CUNNINGHAM, I., BERMAN, E., COLEMAN, M., LANGLEBEN, A., COLVIN, O.M., FUKS, Z., O'REILLY, R. & CLARKSON, B. (1988). Autologous bone marrow transplantation for patients with poor-prognosis lymphoma. *J. Clin. Oncol.*, **6**, 1303-1313.

HJORTH, M., HELLQUIST, L., HOLMBERG, E., MAGNUSSON, B., RODJER, S. & WESTIN, J. (1990). Initial treatment in multiple myeloma: no advantage of multidrug chemotherapy over melphalan-prednisone. The Myeloma Group of Western Sweden. *Br. J. Haematol.*, **74**, 185-191.

JAGANNATH, S., BARLOGIE, B., DICKE, K., ALEXANIAN, R., ZAGARS, G., CHESON, B., LEMAISTRE, F.C., SMALLWOOD, L., PRUITT, K. & DIXON, D.O. (1990). Autologous bone marrow transplantation in multiple myeloma: identification of prognostic factors. *Blood*, **76**, 1860-1866.

JAGANNATH, S., VESOLE, D.H., GLENN, L., CROWLEY, J. & BARLOGIE, B. (1992). Low-risk intensive therapy for multiple myeloma with combined autologous bone marrow and blood stem cell support. *Blood*, **80**, 1666-1672.

JERNBERG-WIKLUND, H., PETTERSSON, M. & NILSSON, K. (1991). Recombinant interferon-gamma inhibits the growth of IL-6-independent human multiple myeloma cell lines in vitro. *Eur. J. Haematol.*, **46**, 231-239.

KLEIN, B., ZHANG, X.G., JOURDAN, M. & BATAILLE, R. (1990). Interleukin-6 is a major myeloma cell growth factor in vitro and in vivo especially in patients with terminal disease. *Curr. Topics Microbiol. Immunol.*, **166**, 23-31.

KLEIN, B., WIJDENES, J., ZHANG, X.G., JOURDAN, M., BOIRON, J.M., BROCHIER, J., LIAUTARD, J., MERLIN, M., CLEMENT, C., MOREL-FOURNIER, B., LU, Z.Y., MANNONI, P., SANY, J. & BATAILLE, R. (1991). Murine anti-interleukin-6 monoclonal antibody therapy for a patient with plasma cell leukemia. *Blood*, **78**, 1198-1204.

LINDAHL, P., LEARY, P. & GRESSER, I. (1972). Enhancement by interferon of the specific cytotoxicity of sensitized lymphocytes. *Proc. Natl Acad. of Sci. USA*, **69**, 721-725.

LINDAHL, P., GRESSER, I., LEARY, P. & TOVEY, M. (1976). Interferon treatment of mice: enhanced expression of histocompatibility antigens on lymphoid cells. *Proc. Nat. Acad. Sci. USA*, **73**, 1284-1287.

LUDWIG, H., NACHBAUR, D.M., FRITZ, E., KRAINER, M. & HUBER, H. (1991*a*). Interleukin-6 is a prognostic factor in multiple myeloma. *Blood*, **77**, 2794-2795.

LUDWIG, H., COHEN, A.M., HUBER, H., NACHBAUR, D., JUNGI, W.F., SENN, H., GUNCZLER, P., SCHULLER, J., ECKHARDT, S., SEEWANN, H.L., CAVALLI, F., FRITZ, E. & MICKSCHE, M. (1991*b*). Interferon alfa-2b with VMCP compared to VMCP alone for induction and interferon alfa-2b compared to controls for remission maintenance in multiple myeloma: interim results. *Eur. J. Cancer*, **27** (Suppl. 4), 40-45.

MCELWAIN, T.J. & POWLES, R.L. (1983). High-dose intravenous melphalan for plasma-cell leukemia and myeloma. *Lancet*, **ii**, 822-824.

MACLENNAN, I.C., CHAPMAN, C., DUNN, J. & KELLY, K. (1992). Combined chemotherapy with ABCM versus melphalan for treatment of myelomatosis. The Medical Research Council Working Party for Leukaemia in Adults. *Lancet*, **339**, 200-205.

MANDELLI, F., AVVISATI, G., AMADORI, S., BOCCADORO, M., GERNONE, A., LAUTA, V.M., MARMONT, F., PETRUCCI, M.T., TRIBALTO, M., VEGNA, M.L., DAMMACCO, F. & PILERI, A. (1990). Maintenance treatment with recombinant interferon alfa-2b in patients with multiple myeloma responding to conventional induction chemotherapy. *N. Engl. J. Med.*, **322**, 1430-1434.

MELLSTEDT, H., AHRE, A., BJORKHOLM, M., HOLM, G., JOHANSSON, B. & STRANDER, H. (1979). Interferon therapy in myelomatosis. *Lancet*, **i**, 245-247.

MRC (1971). Myelomatosis: comparison of melphalan and cyclophosphamide therapy. *Br. Med. J.*, **i**, 640-641.

MRC (1980). Treatment comparisons in the third MRC myelomatosis trial. Medical Research Council's Working Party on Leukaemia in Adults. *Br. J. Cancer*, **42**, 823-830.

OHNO, R. & KIMURA, K. (1986). Treatment of multiple myeloma with recombinant α-interferon. *Cancer*, **57**, 1685-1688.

OKEN, M.M., KYLE, R.A., GREIPP, P.R., KAY, N.E., TSIATIS, A. & O'CONNELL, M.J. (1992). Possible survival benefit with chemotherapy plus interferon (rIFNα2) in the treatment of multiple myeloma. *ASCO Abstracts*, **11**, 358.

OMEDE, P., BOCCADORO, M., GALLONE, G., FRIERI, R., BATTAGLIO, S., REDOGLIA, V. & PILERI, A. (1990). Multiple myeloma: increased circulating lymphocytes carrying plasma cell-associated antigens as an indicator of poor survival. *Blood*, **76**, 1375-1379.

OSTERBORG, A. & MELLSTEDT, H. (1991). The mechanisms of action and the role of alpha interferon in the therapy of myeloma. In *Interferons: Mechanisms of action and Role in Cancer Therapy*. D. Crowther (ed.) pp. 25-31. Springer: Berlin.

OSTERBORG, A., AHRE, A., BJORKHOLM, M., BJOREMAN, M., BRENNING, G., GAHRTON, G., GYLLENHAMMAR, H., JOHANSSON, B., JULIUSSON, G., JARNMARK, M., KILLANDER, A., KIMBY, E., LERNER, R., NILSSON, B., PAUL, C., SIMONSSON, B., STALFELT, A.M., STRANDER, H., SMEDMYR, B., SVEDMYR, E., UDEN, A.M., WADMAN, B., WEDELIN, C. & MELLSTEDT, H. (1989). Alternating combination chemotherapy (VMCP/VBAP) is not superior to melphalan/prednisone in the treatment of multiple myeloma patients stage III-a randomized study from MGCS. *Eur. J. Haematol.*, **43**, 54-62.

OSTERBORG, A., BJORKHOLM, M., BJOREMAN, M., BRENNING, G., CARLSON, K., CELSING, F., GAHRTON, G., GRIMFORS, G., GYLLENHAMMAR, H., HAST, R., JOHANSSON, B., JULIUSSON, G., JARNMARK, M., KIMBY, E., LERNER, R., LINDER, O., MERK, K., NILSSON, B., OHRLING, M., PAUL, C., SIMONSSON, B., SVEDMYR, B., SVEDMYR, E., STALFELT, A.M., STRANDER, H., UDEN, A.M., OSBY, E & MELLSTEDT, H. (1993). Natural interferon-α in combination with melphalan/prednisone versus melphalan/prednisone in the treatment of multiple myeloma stages II and III: a randomized study from the Myeloma Group of Central Sweden. *Blood*, **81**, 1428–1434.

PAVLOVSKY, S., CORRADO, C., SANTARELLI, M.T., SASLAVSKY, J., CAVAGNARO, F., PALAU, M., DE TAZANOS-PINTO, M., HUBERMAN, A. & LEIN, J.M. (1988). An update of two randomized trials in previously untreated multiple myeloma comparing melphalan and prednisone versus three- and five-drug combinations; an Argentine Group for the Treatment of Acute Leukemia Study. *J. Clin. Oncol.*, **6**, 769–775.

PEEST, D., DEICHER, H., COLDEWEY, R., SCHMOLL, H.J. & SCHEDEL, I. (1988). Induction and maintenance therapy in multiple myeloma: a multicenter trial of MP versus VCMP. *Eur. J. Cancer Clin. Oncol.*, **24**, 1061–1067.

PHILIP, T., ARMITAGE, J., SPITZER, G., CHAUVIN, F., JAGANNATH, S., CAHN, J.-Y., COLOMBAT, P., GOLDSTONE, A., GORIN, N., FLESH, M., LAPORTE, J.-P., MARANINCHI, D., PICO, J., BOSLY, A., ANDERSON, C., SCHOTS, R., BIRON, P., CABANILLAS, F. & DICKE, K. (1987). High-dose therapy and autologous bone marrow transplantation after failure of conventional chemotherapy in adults with intermediate-grade or high-grade non-Hodgkin's lymphoma. *N. Engl. J. Med.*, **316**, 1493–1498.

PORTIER, M., ZHANG, X.G., CARON, E., LU, Z.Y., BATAILLE, R. & KLEIN, B. (1993). Gamma-interferon in multiple myeloma: inhibition of interleukin-6-dependent myeloma cell growth and downregulation of IL-6 receptor expression in vitro. *Blood*, **81**, 3076–3082.

REECE, D.E., BARNETT, M.J., CONNORS, J.M., KLINGEMANN, H.G., O'REILLY, S.E., SHEPHERD, J.D., SUTHERLAND, H.J. & PHILLIPS, G.L. (1993). Treatment of multiple myeloma with intensive chemotherapy followed by autologous BMT using marrow purged with 4-hydroperoxycyclophosphamide. *Bone Marrow Transplant.*, **11**, 139–146.

REIFFERS, J., MARIT, G. & BOIRON, J.M. (1989). Autologous blood stem cell transplantation in high-risk multiple myeloma. *Br. J. Haematol.*, **72**, 296–297.

RIVERS, S.L. & PATNO, M.E. (1969). Cyclophosphamide vs melphalan in treatment of plasma cell myeloma. *J. Am. Med. Assoc.*, **207**, 1328–1334.

SALMON, S.E. & CROWLEY, J. (1992). Impact of glucocorticoids and interferon on outcome in multiple myeloma. *ASCO Abstracts*, **11**, 316.

SALMON, S.E., HAUT, A., BONNET, J.D., AMARE, M., WEICK, J.K., DURIE, B.G. & DIXON, D.O. (1983*a*). Alternating combination chemotherapy and levamisole improves survival in multiple myeloma: a Southwest Oncology Group Study. *J. Clin. Oncol.*, **1**, 453–461.

SALMON, S.E., DURIE, B.G., YOUNG, L., LIU, R.M., TROWN, P.W. & STEBBING, N. (1983*b*). Effects of cloned human leukocyte interferons in the human tumor stem cell assay. *J. Clin. Oncol.*, **1**, 217–225.

SALMON, S.E., GROGAN, T.M., MILLER, T., SCHEPER, R. & DALTON, W.S. (1989). Prediction of doxorubicin resistance in vitro in myeloma, lymphoma, and breast cancer by P-glycoprotein staining. *J. Nat. Cancer Instit.*, **81**, 696–701.

SALMON, S.E., DALTON, W.S., GROGAN, T.M., PLEZIA, P., LEHNERT, M., ROE, D.J. & MILLER, T.P. (1990). Multidrug-resistant myeloma: laboratory and clinical effects of verapamil as a chemosensitizer. *Blood*, **78**, 44–50.

SAMSON, D., GAMINARA, E., NEWLAND, A., VAN DE PETTE, J., KEARNEY, J., MCCARTHY, D., JOYNER, M., ASTON, L., MITCHELL, T., HAMON, M., BARRETT, A.J. & EVANS, M. (1989). Infusion of vincristine and doxorubicin with oral dexamethasone as first-line treatment for multiple myeloma. *Lancet*, **ii**, 882–885.

SELBY, P.J., MCELWAIN, T.J., NANDI, A.C., PERREN, T.J., POWLES, R.L., TILLYER, C.R., OSBORNE, R.J., SLEVIN, M.L. & MALPAS, J.S. (1987). Multiple myeloma treated with high dose intravenous melphalan. *Br. J. Haematol.*, **66**, 55–62.

SIDELL, N., TAGA, T., HIRANO, T., KISHIMOTO, T. & SAXON, A. (1991). Retinoic acid-induced growth inhibition of a human myeloma cell line via down-regulation of IL-6 receptors. *J. Immunol.*, **146**, 3809–3814.

SONNEVELD, P., DURIE, B.G.M., LOKHORST, H.M., MARIE, J.-P., SOLBU, G., SUCIU, S., ZITTOUN, R., LOWENBERG, B. & NOOTER, K. (1992). Modulation of multidrug-resistant multiple myeloma by cyclosporin. *Lancet*, **340**, 255–259.

TO, L.B., ROBERTS, M.M., HAYLOCK, D.N., DYSON, P.G., BRANFORD, A.L., THORP, D., HO, J.Q., DART, G.W., HORVATH, N. & DAVY, M.L. (1992). Comparison of haematological recovery times and supportive care requirements of autologous recovery phase peripheral blood stem cell transplants, autologous bone marrow transplants and allogenic bone marrow transplants. *Bone Marrow Transplant.*, **9**, 277–284.

WAGSTAFF, J., LOYNDS, P. & SCARFFE, J.H. (1985). Phase II study of rDNA human alpha-2 interferon in multiple myeloma. *Cancer Treat. Rep.*, **69**, 495–498.

WELANDER, C.E., MORGAN, T.M. & HOMESLEY, H.D. (1985). Combined recombinant human interferon alpha-2 and cytotoxic agents studies in the clonogenic assay. *Int. J. Cancer*, **35**, 721–729.

WESTIN, J., CORTELEZZI, A., HJORTH, M., RODJER, S., TURESSON, I. & ZADOR, G. (1991). Interferon therapy during the plateau phase of multiple myeloma: an update of the Swedish study. *Eur. J. Cancer*, **27** (Suppl.), 4.

ZHANG, X.G., KLEIN, B. & BATAILLE, R. (1989). Interleukin-6 is a potent myeloma-cell growth factor in patients with aggressive multiple myeloma. *Blood*, **74**, 11–13.